# Elevated Pertussis Reporting in Response to 2011–2012 Outbreak, New York City, New York, USA 

**DOI:** 10.3201/eid2206.151514

**Published:** 2016-06

**Authors:** Robert J. Arciuolo, Jennifer B. Rosen, Jane R. Zucker

**Affiliations:** Centers for Disease Control and Prevention/Council of State and Territorial Epidemiologists Applied Epidemiology Fellowship, Atlanta, Georgia, USA (R.J. Arciuolo);; New York City Department of Health and Mental Hygiene, New York, New York, USA (R.J. Arciuolo, J.B. Rosen, J.R. Zucker);; Centers for Disease Control and Prevention, Atlanta (J.R. Zucker)

**Keywords:** Bordetella pertussis, surveys, surveillance, bacteria, disease notification, communicable disease control, New York City, United States, New York, pertussis, whooping cough

**To the Editor:** Pertussis is a highly communicable, acute bacterial respiratory infection caused by *Bordetella pertussis*. In the United States, the incidence of pertussis declined dramatically after pertussis-containing vaccine was introduced in the 1940s (*1**,**2*). However, a resurgence of disease results in widespread outbreaks of pertussis nationally (*3*).

Beginning in August 2011, an outbreak of pertussis occurred in New York City (NYC), New York, USA. Reported pertussis incidence by month peaked in December 2011 (1.03 cases/100,000 persons) and remained above the baseline average monthly incidence of 0.11 cases/100,000 persons until February 2013. We hypothesized that provider awareness and altered practices after the start of the outbreak contributed to the sustained elevation in reported pertussis incidence.

To test this hypothesis, we surveyed NYC providers to assess their awareness of the outbreak, their consideration of pertussis in symptomatic patients, and the type and frequency of diagnostic testing ordered. The survey (available on request) was designed in FeedbackServer 5 (University of Massachusetts, Worcester, MA, USA; https://w3.umassmed.edu/fs/FeedbackServer/help/feedbackserver.htm) and consisted of 20 questions that required ≈5 minutes to complete by using a Web link. We distributed the survey in January 2013 to providers through 3 health department email lists: the NYC Health Alert Network, the Citywide Immunization Registry, and the Primary Care Information Project. The lists included ≈30,000 email addresses that were not mutually exclusive and that included nonmedical providers.

Through March 7, 2013, we received 1,316 responses; 887 (67%) were excluded from analyses for >1 reason: respondent did not complete all survey questions (74%); respondent did not practice in a hospital or outpatient facility (31%); respondent indicated that his or her primary facility was located outside NYC (6%); or response was a duplicate (<1%). Of the 429 (33%) responses included in our analyses, 69% of respondents served adults and 54% served children (23% served both adults and children); 38% practiced in a hospital, and 81% practiced in an outpatient setting (18% practiced in both hospital and outpatient settings).

Respondents were asked if and how they were aware of the pertussis outbreak; 84% reported previous awareness of the outbreak. The top reported sources contributing to respondents’ outbreak awareness included health advisory alerts (local [80%], state [36%], and national [40%]); media reports (36%); and discussion with colleagues (29%).

In addition, respondents were asked how likely they were to consider pertussis infection in patients with prolonged cough before 2012 and currently. Reported consideration of pertussis before 2012 varied: 35% of respondents were likely or very likely to consider pertussis, 33% were somewhat likely to consider pertussis, 30% were unlikely to consider pertussis, and 3% did not know (unknown). However, 73% of respondents said that they were more likely to consider pertussis at the time of the survey than before 2012. The top reported sources contributing to increased consideration of pertussis mirrored those contributing to outbreak awareness.

Respondents were last asked to assess the type and frequency of diagnostic testing they used before and since 2012 (Table). Most (66%) respondents indicated that they did not perform diagnostic testing for pertussis before 2012. Among the 34% who tested for pertussis before 2012, the main diagnostic methods used were bacterial culture (46%) and PCR (45%). However, 12% of respondents indicated that they had changed the type of diagnostic test they used beginning in 2012; among these respondents, 33% were more likely to use pertussis culture and 63% were more likely to use PCR or to use culture and PCR. Of total respondents, 22% indicated that they ordered diagnostic tests more frequently since the beginning of 2012.

**Table:** Provider responses to survey questions related to diagnostic testing for pertussis, New York City, New York, USA*

Questions and responses	No. (%)
Did respondent perform diagnostic tests for pertussis before 2012?	
Did not perform diagnostic tests	282 (66)
Performed ≥1 diagnostic test	147 (34)
Which types of diagnostic tests did respondent perform before 2012?†	
Bacterial culture	68 (46)
PCR	66 (45)
Serology	35 (24)
DFA	41 (28)
Did respondent change the types of diagnostic tests he/she performed since 2012?‡	
Yes	52 (12)
No	269 (63)
Unknown	108 (25)
Which types of diagnostic tests was respondent more likely to perform since 2012?§	
Bacterial culture	17 (33)
PCR	33 (63)
Serology	6 (12)
DFA	5 (10)
No test	3 (6)
Did respondent change the frequency of diagnostic tests he/she performed since 2012?	
More frequent testing	93 (22)
Less frequent testing	14 (3)
Frequency of testing unchanged	231 (54)
No response provided	1 (<1)

*N = 429 except as indicated. DFA, direct fluorescent antibody test. †For 147 respondents who reported performing >1 diagnostic test before 2012. Response choices are not mutually exclusive. ‡For all respondents, including those who reported not performing diagnostic tests before 2012. §For 52 respondents who reported a change in type of diagnostic test performed since 2012. Response choices are not mutually exclusive.

Our investigation has limitations. We could not determine a survey response rate because of extensive overlap of the email lists used, and we lacked access to the lists; the response rate is assumed to be very low. Respondents included in the analysis may not have been representative of the broader NYC provider community. In addition, respondents may not have uniformly interpreted the survey because of the subjective nature of some survey questions, and recall bias may have affected responses. Also, respondent awareness of the outbreak is likely overestimated because the email lists used for survey distribution were used during the outbreak to distribute health alerts. Despite these limitations, our investigation shows the value of Web-based surveys distributed by email to gather information rapidly from a large provider community in a cost-effective and practical manner.

This investigation indicates the importance of provider knowledge and practices for public health surveillance data. High awareness of an outbreak, increased clinical suspicion of pertussis, and increased frequency of diagnostic testing likely contributed to a sustained elevation in pertussis incidence. Advisory alerts and media reports were successful mechanisms for disseminating information to providers during the outbreak and likely altered provider behaviors that contributed to the increase in reported pertussis incidence. Previous reports have documented increased submission of disease notifications after media coverage of health concerns (*4**,**5*). Responses to our survey also highlight how pertussis incidence may be routinely underestimated because providers do not suspect the disease or test for it consistently.
